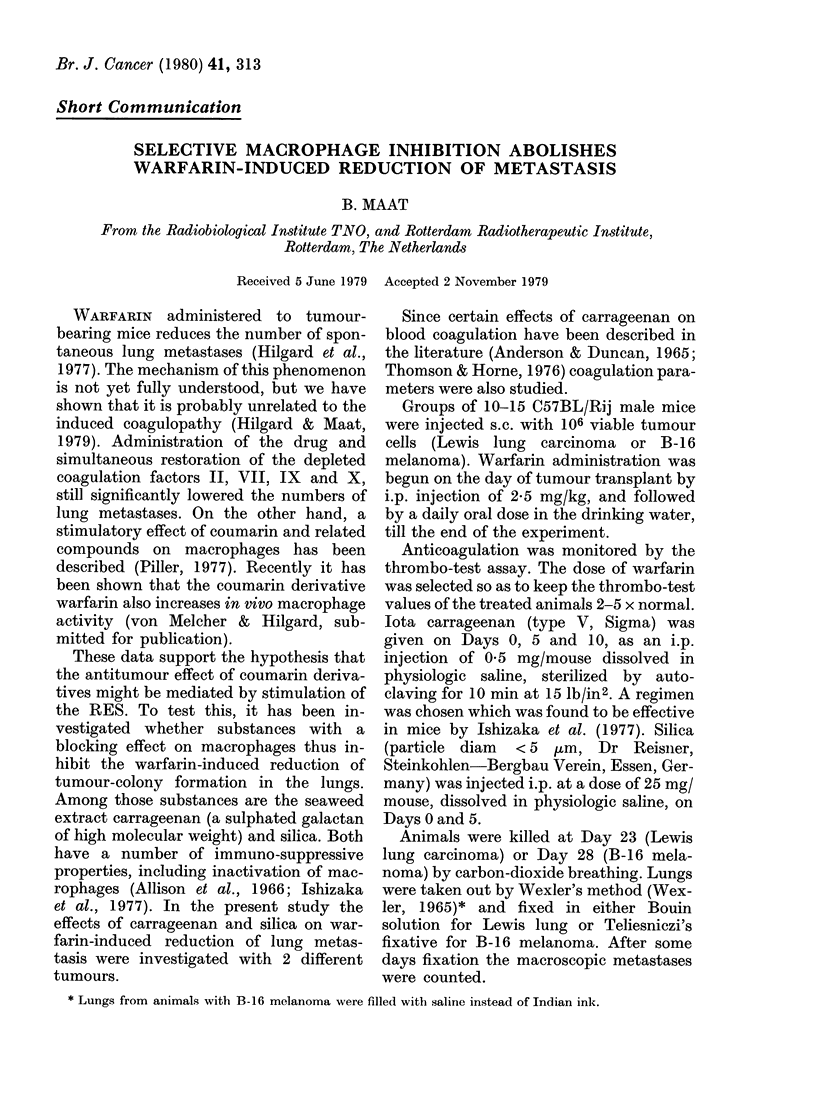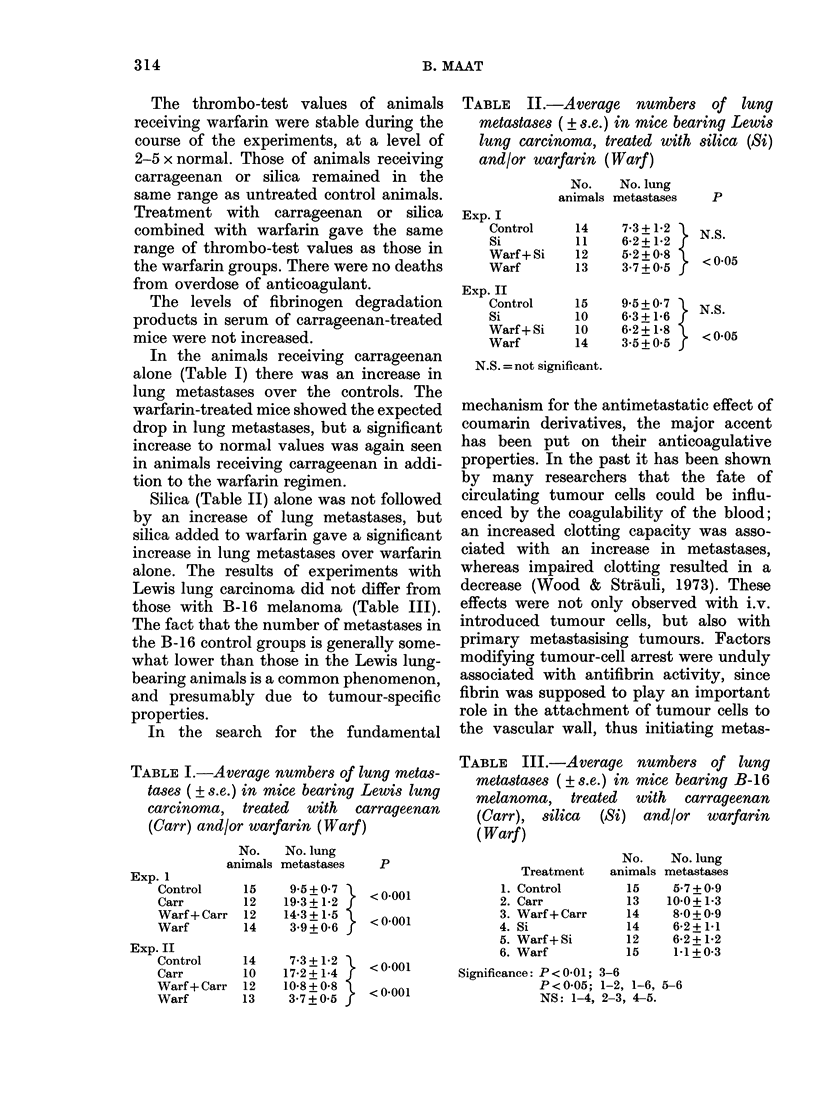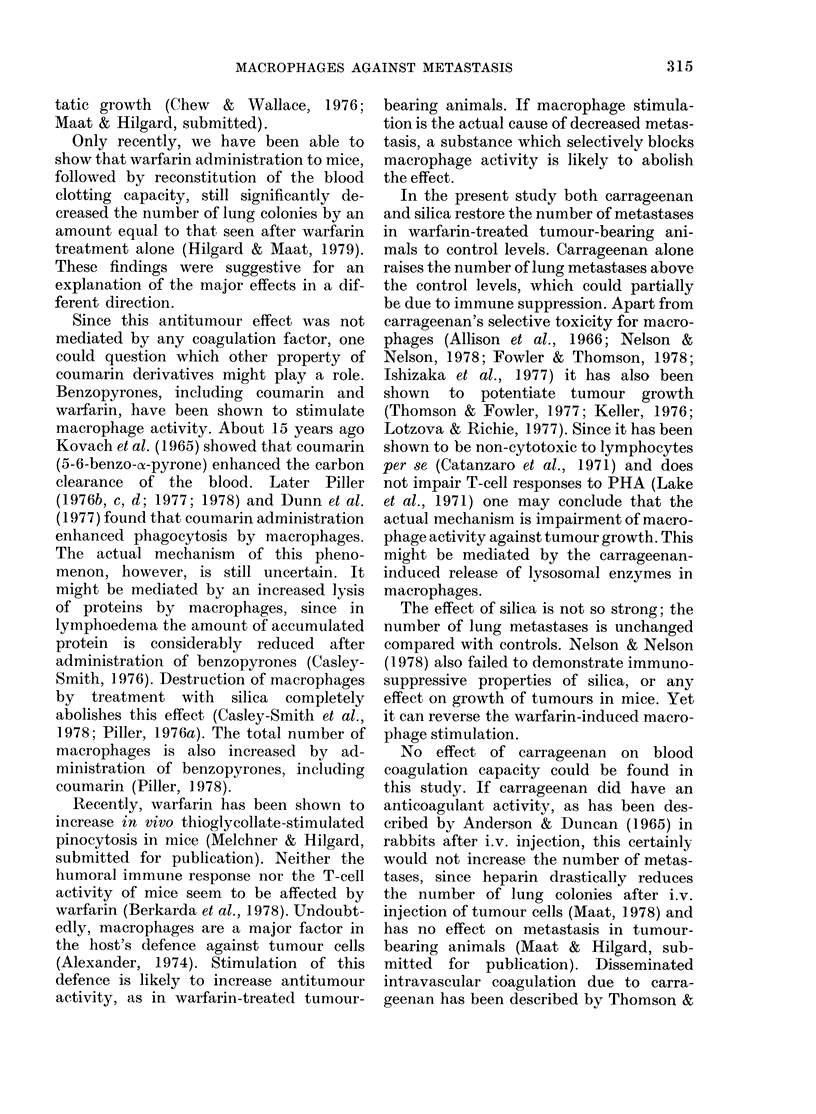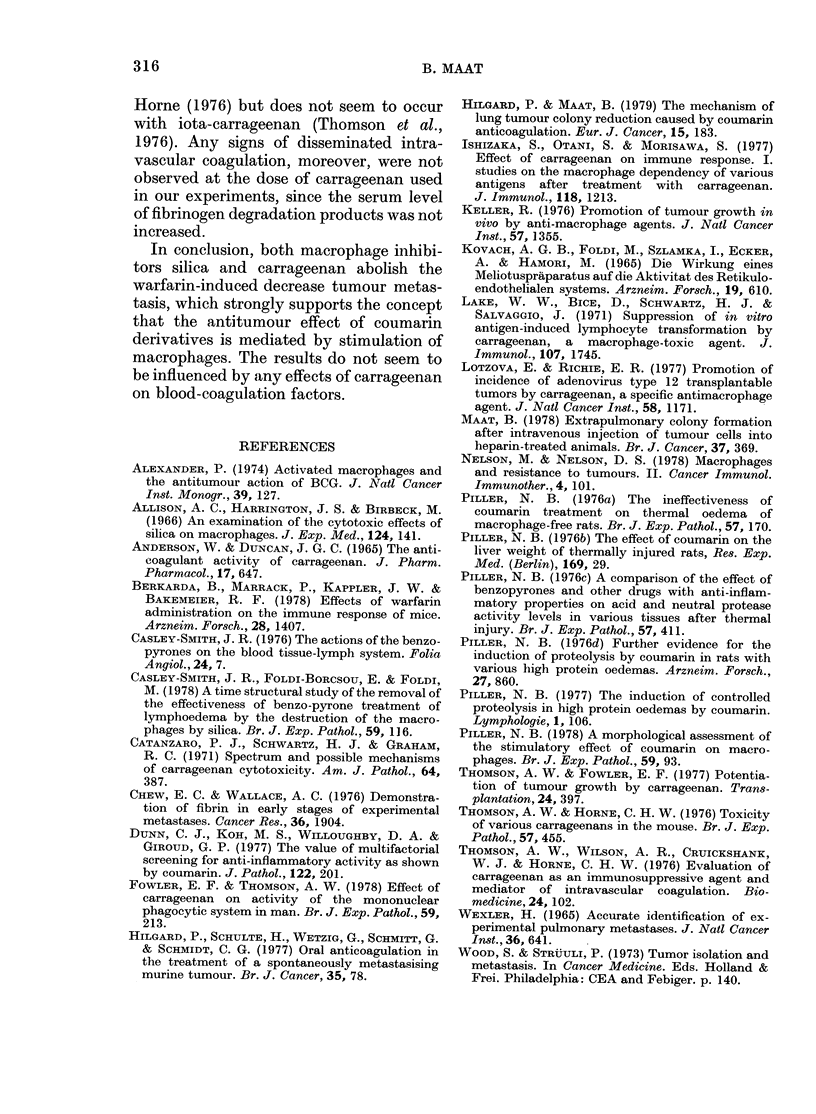# Selective macrophage inhibition abolishes warfarin-induced reduction of metastasis.

**DOI:** 10.1038/bjc.1980.46

**Published:** 1980-02

**Authors:** B. Maat


Br. J. Cancer (1980) 41, 313

***Short Communication***

### SELECTIVE MACROPHAGE INHIBITION ABOLISHES WARFARIN-INDUCED REDUCTION OF METASTASIS

B. MAAT

*From the Radiobiological Institute TNO, and Rotterdam Radiotherapeutic Institute, Rotterdam, The Netherlands*

Received 5 June 1979 Accepted 2 November 1979

WARFARIN administered to tumour-bearing mice reduces the number of spontaneous lung metastases (Hilgard *et al.*, 1977). The mechanism of this phenomenon is not yet fully understood, but we have shown that it is probably unrelated to the induced coagulopathy (Hilgard & Maat, 1979). Administration of the drug and simultaneous restoration of the depleted coagulation factors II, VII, IX and X, still significantly lowered the numbers of lung metastases. On the other hand, a stimulatory effect of coumarin and related compounds on macrophages has been described (Piller, 1977). Recently it has been shown that the coumarin derivative warfarin also increases *in vivo* macrophage activity (von Melcher & Hilgard, submitted for publication).

These data support the hypothesis that the antitumour effect of coumarin derivatives might be mediated by stimulation of the RES. To test this, it has been investigated whether substances with a blocking effect on macrophages thus inhibit the warfarin-induced reduction of tumour-colony formation in the lungs. Among those substances are the seaweed extract carrageenan (a sulphated galactan of high molecular weight) and silica. Both have a number of immuno-suppressive properties, including inactivation of macrophages (Allison *et al.*, 1966; Ishizaka *et al.*, 1977). In the present study the effects of carrageenan and silica on warfarin-induced reduction of lung metastasis were investigated with 2 different tumours.

Since certain effects of carrageenan on blood coagulation have been described in the literature (Anderson & Duncan, 1965; Thomson & Horne, 1976) coagulation parameters were also studied.

Groups of 10–15 C57BL/Rij male mice were injected s.c. with $10^6$ viable tumour cells (Lewis lung carcinoma or B-16 melanoma). Warfarin administration was begun on the day of tumour transplant by i.p. injection of 2·5 mg/kg, and followed by a daily oral dose in the drinking water, till the end of the experiment.

Anticoagulation was monitored by the thrombo-test assay. The dose of warfarin was selected so as to keep the thrombo-test values of the treated animals 2–5 × normal. Iota carrageenan (type V, Sigma) was given on Days 0, 5 and 10, as an i.p. injection of 0·5 mg/mouse dissolved in physiologic saline, sterilized by autoclaving for 10 min at 15 lb/in$^2$. A regimen was chosen which was found to be effective in mice by Ishizaka *et al.* (1977). Silica (particle diam $< 5$ $\mu$m, Dr Reisner, Steinkohlen—Bergbau Verein, Essen, Germany) was injected i.p. at a dose of 25 mg/mouse, dissolved in physiologic saline, on Days 0 and 5.

Animals were killed at Day 23 (Lewis lung carcinoma) or Day 28 (B-16 melanoma) by carbon-dioxide breathing. Lungs were taken out by Wexler's method (Wexler, 1965)* and fixed in either Bouin solution for Lewis lung or Teliesniczi's fixative for B-16 melanoma. After some days fixation the macroscopic metastases were counted.

* Lungs from animals with B-16 melanoma were filled with saline instead of Indian ink.

The thrombo-test values of animals receiving warfarin were stable during the course of the experiments, at a level of 2–5 × normal. Those of animals receiving carrageenan or silica remained in the same range as untreated control animals. Treatment with carrageenan or silica combined with warfarin gave the same range of thrombo-test values as those in the warfarin groups. There were no deaths from overdose of anticoagulant.

The levels of fibrinogen degradation products in serum of carrageenan-treated mice were not increased.

In the animals receiving carrageenan alone (Table I) there was an increase in lung metastases over the controls. The warfarin-treated mice showed the expected drop in lung metastases, but a significant increase to normal values was again seen in animals receiving carrageenan in addition to the warfarin regimen.

Silica (Table II) alone was not followed by an increase of lung metastases, but silica added to warfarin gave a significant increase in lung metastases over warfarin alone. The results of experiments with Lewis lung carcinoma did not differ from those with B-16 melanoma (Table III). The fact that the number of metastases in the B-16 control groups is generally somewhat lower than those in the Lewis lung-bearing animals is a common phenomenon, and presumably due to tumour-specific properties.

In the search for the fundamental mechanism for the antimetastatic effect of coumarin derivatives, the major accent has been put on their anticoagulative properties. In the past it has been shown by many researchers that the fate of circulating tumour cells could be influenced by the coagulability of the blood; an increased clotting capacity was associated with an increase in metastases, whereas impaired clotting resulted in a decrease (Wood & Sträuli, 1973). These effects were not only observed with i.v. introduced tumour cells, but also with primary metastasising tumours. Factors modifying tumour-cell arrest were unduly associated with antifibrin activity, since fibrin was supposed to play an important role in the attachment of tumour cells to the vascular wall, thus initiating metas-

TABLE I.—*Average numbers of lung metastases (± s.e.) in mice bearing Lewis lung carcinoma, treated with carrageenan (Carr) and/or warfarin (Warf)*

| | No. animals | No. lung metastases | P |
|---|---|---|---|
| Exp. 1 | | | |
| Control | 15 | $9.5 \pm 0.7$ | } < 0·001 |
| Carr | 12 | $19.3 \pm 1.2$ | |
| Warf + Carr | 12 | $14.3 \pm 1.5$ | } < 0·001 |
| Warf | 14 | $3.9 \pm 0.6$ | |
| Exp. II | | | |
| Control | 14 | $7.3 \pm 1.2$ | } < 0·001 |
| Carr | 10 | $17.2 \pm 1.4$ | |
| Warf + Carr | 12 | $10.8 \pm 0.8$ | } < 0·001 |
| Warf | 13 | $3.7 \pm 0.5$ | |

TABLE II.—*Average numbers of lung metastases (± s.e.) in mice bearing Lewis lung carcinoma, treated with silica (Si) and/or warfarin (Warf)*

| | No. animals | No. lung metastases | P |
|---|---|---|---|
| Exp. I | | | |
| Control | 14 | $7.3 \pm 1.2$ | } N.S. |
| Si | 11 | $6.2 \pm 1.2$ | |
| Warf + Si | 12 | $5.2 \pm 0.8$ | } < 0·05 |
| Warf | 13 | $3.7 \pm 0.5$ | |
| Exp. II | | | |
| Control | 15 | $9.5 \pm 0.7$ | } N.S. |
| Si | 10 | $6.3 \pm 1.6$ | |
| Warf + Si | 10 | $6.2 \pm 1.8$ | } < 0·05 |
| Warf | 14 | $3.5 \pm 0.5$ | |

N.S. = not significant.

TABLE III.—*Average numbers of lung metastases (± s.e.) in mice bearing B-16 melanoma, treated with carrageenan (Carr), silica (Si) and/or warfarin (Warf)*

| Treatment | No. animals | No. lung metastases |
|---|---|---|
| 1. Control | 15 | $5.7 \pm 0.9$ |
| 2. Carr | 13 | $10.0 \pm 1.3$ |
| 3. Warf + Carr | 14 | $8.0 \pm 0.9$ |
| 4. Si | 14 | $6.2 \pm 1.1$ |
| 5. Warf + Si | 12 | $6.2 \pm 1.2$ |
| 6. Warf | 15 | $1.1 \pm 0.3$ |

Significance: $P < 0.01$; 3–6
$P < 0.05$; 1–2, 1–6, 5–6
NS: 1–4, 2–3, 4–5.

tatic growth (Chew & Wallace, 1976; Maat & Hilgard, submitted).

Only recently, we have been able to show that warfarin administration to mice, followed by reconstitution of the blood clotting capacity, still significantly decreased the number of lung colonies by an amount equal to that seen after warfarin treatment alone (Hilgard & Maat, 1979). These findings were suggestive for an explanation of the major effects in a different direction.

Since this antitumour effect was not mediated by any coagulation factor, one could question which other property of coumarin derivatives might play a role. Benzopyrones, including coumarin and warfarin, have been shown to stimulate macrophage activity. About 15 years ago Kovach *et al.* (1965) showed that coumarin (5-6-benzo-$\alpha$-pyrone) enhanced the carbon clearance of the blood. Later Piller (1976*b*, *c*, *d*; 1977; 1978) and Dunn *et al.* (1977) found that coumarin administration enhanced phagocytosis by macrophages. The actual mechanism of this phenomenon, however, is still uncertain. It might be mediated by an increased lysis of proteins by macrophages, since in lymphoedema the amount of accumulated protein is considerably reduced after administration of benzopyrones (Casley-Smith, 1976). Destruction of macrophages by treatment with silica completely abolishes this effect (Casley-Smith *et al.*, 1978; Piller, 1976*a*). The total number of macrophages is also increased by administration of benzopyrones, including coumarin (Piller, 1978).

Recently, warfarin has been shown to increase *in vivo* thioglycollate-stimulated pinocytosis in mice (Melchner & Hilgard, submitted for publication). Neither the humoral immune response nor the T-cell activity of mice seem to be affected by warfarin (Berkarda *et al.*, 1978). Undoubtedly, macrophages are a major factor in the host's defence against tumour cells (Alexander, 1974). Stimulation of this defence is likely to increase antitumour activity, as in warfarin-treated tumour-bearing animals. If macrophage stimulation is the actual cause of decreased metastasis, a substance which selectively blocks macrophage activity is likely to abolish the effect.

In the present study both carrageenan and silica restore the number of metastases in warfarin-treated tumour-bearing animals to control levels. Carrageenan alone raises the number of lung metastases above the control levels, which could partially be due to immune suppression. Apart from carrageenan's selective toxicity for macrophages (Allison *et al.*, 1966; Nelson & Nelson, 1978; Fowler & Thomson, 1978; Ishizaka *et al.*, 1977) it has also been shown to potentiate tumour growth (Thomson & Fowler, 1977; Keller, 1976; Lotzova & Richie, 1977). Since it has been shown to be non-cytotoxic to lymphocytes *per se* (Catanzaro *et al.*, 1971) and does not impair T-cell responses to PHA (Lake *et al.*, 1971) one may conclude that the actual mechanism is impairment of macrophage activity against tumour growth. This might be mediated by the carrageenan-induced release of lysosomal enzymes in macrophages.

The effect of silica is not so strong; the number of lung metastases is unchanged compared with controls. Nelson & Nelson (1978) also failed to demonstrate immunosuppressive properties of silica, or any effect on growth of tumours in mice. Yet it can reverse the warfarin-induced macrophage stimulation.

No effect of carrageenan on blood coagulation capacity could be found in this study. If carrageenan did have an anticoagulant activity, as has been described by Anderson & Duncan (1965) in rabbits after i.v. injection, this certainly would not increase the number of metastases, since heparin drastically reduces the number of lung colonies after i.v. injection of tumour cells (Maat, 1978) and has no effect on metastasis in tumour-bearing animals (Maat & Hilgard, submitted for publication). Disseminated intravascular coagulation due to carrageenan has been described by Thomson &

Horne (1976) but does not seem to occur with iota-carrageenan (Thomson *et al.*, 1976). Any signs of disseminated intravascular coagulation, moreover, were not observed at the dose of carrageenan used in our experiments, since the serum level of fibrinogen degradation products was not increased.

In conclusion, both macrophage inhibitors silica and carrageenan abolish the warfarin-induced decrease tumour metastasis, which strongly supports the concept that the antitumour effect of coumarin derivatives is mediated by stimulation of macrophages. The results do not seem to be influenced by any effects of carrageenan on blood-coagulation factors.

### REFERENCES

ALEXANDER, P. (1974) Activated macrophages and the antitumour action of BCG. *J. Natl Cancer Inst. Monogr.*, **39**, 127.

ALLISON, A. C., HARRINGTON, J. S. & BIRBECK, M. (1966) An examination of the cytotoxic effects of silica on macrophages. *J. Exp. Med.*, **124**, 141.

ANDERSON, W. & DUNCAN, J. G. C. (1965) The anticoagulant activity of carrageenan. *J. Pharm. Pharmacol.*, **17**, 647.

BERKARDA, B., MARRACK, P., KAPPLER, J. W. & BAKEMEIER, R. F. (1978) Effects of warfarin administration on the immune response of mice. *Arzneim. Forsch.*, **28**, 1407.

CASLEY-SMITH, J. R. (1976) The actions of the benzopyrones on the blood tissue-lymph system. *Folia Angiol.*, **24**, 7.

CASLEY-SMITH, J. R., FOLDI-BORCSOU, E. & FOLDI, M. (1978) A time structural study of the removal of the effectiveness of benzo-pyrone treatment of lymphoedema by the destruction of the macrophages by silica. *Br. J. Exp. Pathol.*, **59**, 116.

CATANZARO, P. J., SCHWARTZ, H. J. & GRAHAM, R. C. (1971) Spectrum and possible mechanisms of carrageenan cytotoxicity. *Am. J. Pathol.*, **64**, 387.

CHEW, E. C. & WALLACE, A. C. (1976) Demonstration of fibrin in early stages of experimental metastases. *Cancer Res.*, **36**, 1904.

DUNN, C. J., KOH, M. S., WILLOUGHBY, D. A. & GIROUD, G. P. (1977) The value of multifactorial screening for anti-inflammatory activity as shown by coumarin. *J. Pathol.*, **122**, 201.

FOWLER, E. F. & THOMSON, A. W. (1978) Effect of carrageenan on activity of the mononuclear phagocytic system in man. *Br. J. Exp. Pathol.*, **59**, 213.

HILGARD, P., SCHULTE, H., WETZIG, G., SCHMITT, G. & SCHMIDT, C. G. (1977) Oral anticoagulation in the treatment of a spontaneously metastasising murine tumour. *Br. J. Cancer*, **35**, 78.

HILGARD, P. & MAAT, B. (1979) The mechanism of lung tumour colony reduction caused by coumarin anticoagulation. *Eur. J. Cancer*, **15**, 183.

ISHIZAKA, S., OTANI, S. & MORISAWA, S. (1977) Effect of carrageenan on immune response. I. studies on the macrophage dependency of various antigens after treatment with carrageenan. *J. Immunol.*, **118**, 1213.

KELLER, R. (1976) Promotion of tumour growth *in vivo* by anti-macrophage agents. *J. Natl Cancer Inst.*, **57**, 1355.

KOVACH, A. G. B., FOLDI, M., SZLAMKA, I., ECKER, A. & HAMORI, M. (1965) Die Wirkung eines Meliotuspräparatus auf die Aktivitat des Retikulo-endothelialen systems. *Arzneim. Forsch.*, **19**, 610.

LAKE, W. W., BICE, D., SCHWARTZ, H. J. & SALVAGGIO, J. (1971) Suppression of *in vitro* antigen-induced lymphocyte transformation by carrageenan, a macrophage-toxic agent. *J. Immunol.*, **107**, 1745.

LOTZOVA, E. & RICHIE, E. R. (1977) Promotion of incidence of adenovirus type 12 transplantable tumors by carrageenan, a specific antimacrophage agent. *J. Natl Cancer Inst.*, **58**, 1171.

MAAT, B. (1978) Extrapulmonary colony formation after intravenous injection of tumour cells into heparin-treated animals. *Br. J. Cancer*, **37**, 369.

NELSON, M. & NELSON, D. S. (1978) Macrophages and resistance to tumours. II. *Cancer Immunol. Immunother.*, **4**, 101.

PILLER, N. B. (1976*a*) The ineffectiveness of coumarin treatment on thermal oedema of macrophage-free rats. *Br. J. Exp. Pathol.*, **57**, 170.

PILLER, N. B. (1976*b*) The effect of coumarin on the liver weight of thermally injured rats, *Res. Exp. Med.* (*Berlin*), **169**, 29.

PILLER, N. B. (1976*c*) A comparison of the effect of benzopyrones and other drugs with anti-inflammatory properties on acid and neutral protease activity levels in various tissues after thermal injury. *Br. J. Exp. Pathol.*, **57**, 411.

PILLER, N. B. (1976*d*) Further evidence for the induction of proteolysis by coumarin in rats with various high protein oedemas. *Arzneim. Forsch.*, **27**, 860.

PILLER, N. B. (1977) The induction of controlled proteolysis in high protein oedemas by coumarin. *Lymphologie*, **1**, 106.

PILLER, N. B. (1978) A morphological assessment of the stimulatory effect of coumarin on macrophages. *Br. J. Exp. Pathol.*, **59**, 93.

THOMSON, A. W. & FOWLER, E. F. (1977) Potentiation of tumour growth by carrageenan. *Transplantation*, **24**, 397.

THOMSON, A. W. & HORNE, C. H. W. (1976) Toxicity of various carrageenans in the mouse. *Br. J. Exp. Pathol.*, **57**, 455.

THOMSON, A. W., WILSON, A. R., CRUICKSHANK, W. J. & HORNE, C. H. W. (1976) Evaluation of carrageenan as an immunosuppressive agent and mediator of intravascular coagulation. *Biomedicine*, **24**, 102.

WEXLER, H. (1965) Accurate identification of experimental pulmonary metastases. *J. Natl Cancer Inst.*, **36**, 641.

WOOD, S. & STRÜULI, P. (1973) Tumor isolation and metastasis. In *Cancer Medicine*. Eds. Holland & Frei. Philadelphia: CEA and Febiger. p. 140.